# Trends among patients with endometriosis over a 7-year period and the impact of the COVID-19 pandemic: experience from an academic high-level endometriosis centre in Germany

**DOI:** 10.1007/s00404-022-06730-x

**Published:** 2022-09-07

**Authors:** Lucia Keilmann, Susanne Beyer, Sarah Meister, Magdalena Jegen, Christina Buschmann, Lennard Schröder, Simon Keckstein, Udo Jeschke, Alexander Burges, Sven Mahner, Fabian Trillsch, Bernd Kost, Thomas Kolben

**Affiliations:** 1 Department of Gynaecology and Obstetrics, University Hospital, LMU Munich, Marchioninistr. 15, 81377 Munich, Germany; 2 Department of Gynaecology and Obstetrics, University Hospital Augsburg, 86156 Augsburg, Germany

**Keywords:** Endometriosis, Awareness, COVID-19, Treatment delay

## Abstract

**Purpose:**

Endometriosis is known to be an underestimated disease. Lately the awareness of the disease seems to have improved. Aim of this analysis is to provide an overview of the development of treatment of patients diagnosed with endometriosis. This includes a special scope on implications of the COVID-19 pandemic since in multiple settings postponed treatments resulting in negative impact on prognosis were reported.

**Materials and methods:**

We analysed the development of numbers of patients treated for endometriosis in an academic centre within a 7-year period, 01/2015–12/2021, performing a systematic analysis of ICD-10-Codes from our computer system used in clinical routine.

**Results:**

Treatment numbers increased over the past 7 years, i.e., 239 treated cases in 2015 vs. 679 in 2021.

Following restrictions for outpatient evaluation and surgical capacity at our centre, during COVID-19 pandemic the numbers of treated patients were reduced, especially in the first lockdown period (03/22/2020–05/05/2020 vs. same period in 2019: outpatient clinic (9 vs. 36; *p* < 0.001), patients surgically treated (27 vs. 52; *p* < 0,001)). The comparison of 2020 to 2019 showed a reduction in April 2020 of − 37% in outpatient department and up to − 90% for surgically treated patients. Comparing to 2019, we found a reduction of surgical interventions in 2020 by − 9% and an increase by 83% in 2021.

**Conclusions:**

Raising numbers of patients treated for endometriosis point to a new awareness for the disease. After the decline during the lockdown period numbers raised again, leading to a delay, but not an omission of treatment. A certified endometriosis centre with established and well-organized structures is required to improve not only treatment results but also quality of life of those affected.

## What does this study add to the clinical work

**Table:** 

The alertness towards endometriosis and the need for its diagnosis and treatment in order to reduce the impact on the patients’ quality of life has been increasing in recent years. Due to COVID restrictions a decrease in treatment numbers of patients with endometriosis was noted during the lockdown-period but could be more than compensated in the following period.	

## Introduction

Endometriosis is one of the most common, if not the most common benign gynaecological disease [1]. Data show that up to 10% of women in the reproductive age suffer from endometriosis. Up to 40–70% of patients with an unfulfilled wish to have children and chronic pelvic pain are diagnosed with endometriosis [2]. Additional symptoms include pelvic pain, dysmenorrhoea, dysuria, dyschezia and dyspareunia [3].

Reports show a long duration from the first symptom to diagnosis, varying from country to country. Focussing on Austria and Germany Hudelist et al. outlined an interval of up to 10 years in their survey and reported that three quarters of the patients with endometriosis were diagnosed incorrectly at least once [4].

The European Endometriosis League (EEL) was founded in 2005 and is directly linked to the German “Stiftung Endometriose Forschung” (SEF) [5]. Its primary goal is to work on this dilemma and to promote awareness for endometriosis and improve diagnosis and therapy of this disease. This is done by public relations, support of research and education of the medical staff. The SEF also closely cooperates the Endometriose-Vereinigung Germany e.V., which is a self-help organisation by and for patients suffering from endometriosis and acting nationwidely. On behalf of SEF and EEL, EuroEndoCert offers certification of centres specialized on endometriosis in Europe [6–8].

Simoens et al. showed the high impact caused by endometriosis and its symptoms such as reduced quality of life, inability to work and severe pain on an individual level on the one hand, as well as high costs for the health care systems on the other hand, caused by surgery, hospitalization and physician visits [9]. These average annual costs were calculated by Prast et al. for Austria exemplarily, identifying in-patient care and loss of productivity as financially most important [10].

On December 31st, 2019 the WHO was informed of cases with pneumonia caused by a virus unknown at this time, which was later named SARS-CoV-2, the severe acute respiratory syndrome corona virus 2. At the end of January 2020, the WHO declared the “novel coronavirus (2019-nCoV) outbreak a public health emergency of international concern (PHEIC), WHO's highest level of alarm” and in March 2020, WHO announced a global pandemic [11, 12].

Worldwide different strategies were used to cope with the immense number of patients suffering from COVID-19. In Germany, the first general lockdown was proclaimed from March 22nd until May 5th 2020. Due to social distancing restrictions and to save capacities, appointments in outpatient departments and planned surgeries were postponed, which had high implications for every patient. Patients with cancer missed their routine check-ups. There are concerns and reports about the postponed diagnosis of various types of cancer [13–15].

Although endometriosis is not a disease with imminent threat for patient´s life, it has an important impact on quality of life of patients diagnosed and especially those who are not diagnosed, but suffer from the heavy implications caused by endometriosis and its symptoms [16].

Since diagnosis and treatment demand a high amount of the patient’s and health care provider’s time, there is a high risk that endometriosis will not receive sufficient attention during the COVID-19 pandemic, because there is no significant lobby and not enough significant clinical interest [17]. To fill the up-coming gap, advice on self-management strategies to fight endometriosis has been published when clinical help is lacking [18]. Furthermore, eHealth programs tried to fill this gap of direct interaction for example with online consultations. It was shown that they can provide a reasonable alternative [19].

The purpose of this study was to evaluate the trend of treatment of patients with endometriosis over a 7-year period at a tertiary academic gynaecological centre in southern Germany. Since there have been serious concerns that patients diagnosed with endometrioses receive less help and treatment, we investigated the implications for diagnosis and treatment of this benign disease during the COVID-19 pandemic in a second step.

Methods

A chart review to identify eligible patients was performed. Patients with treatment for endometriosis between 01/2015 and 12/2021 at the University Hospital LMU Munich were included. We calculated differences through the years and changes between the COVID-19 period and the interval of the last few years for outpatient and inpatient cases.

The centre for endometriosis of the LMU Munich was certified by the SEF in 2013 for the first time.

In 2019 the certification for the highest level of patient care was achieved including a broad arrangement with cooperations (surgery, urology, reproductive medicine, pain therapy, psychology team, dietary counselling, etc.). This level also demands regular educational and skill trainings for the medical staff of the endometriosis team, furthermore participation in research on endometriosis.

For this analysis all patients diagnosed with endometriosis at the Department of Obstetrics and Gynaecology of the University Hospital, LMU Munich, between January 2015 and December 2021 were included to evaluate the development of patients with endometriosis treated in our outpatient as well as our surgical department over the last 7 years and to compare the first 2 years of the COVID-19-pandemic to the preceding year 2019. Focussing on the first lockdown period dated from March 22nd until May 5th 2020, patient numbers treated in outpatient department and with surgery were compared to the same period in 2019 (03/22/2020–05/05/2020 vs. 03/22/2019–05/05/2019).The data was collected in a systematic analysis of ICD-10-Codes from our computer system used in clinical routine KAS–SAP. In this analysis we included the ICD-10-Code N80.0 (adenomyosis), N80.1 (endometriosis of the ovary), N80.2 (endometriosis of the tubes), N80.3 (peritoneal endometriosis), N80.4 (endometriosis of the vagina or found in the rectovaginal septum), N80.5 (endometriosis of the intestine), N80.6 (endometriosis of scar or skin), N80.6 (endometriosis: other including thoracic endometriosis) and 80.9 (endometriosis non specified).

Microsoft Excel 2016 and SPSS Statistics 28 (IBM, Chicago, IL, USA) were used for statistical analysis. Significance was tested using the chi square test, *α* = 0.05.

## Results

**Fig. 1:**
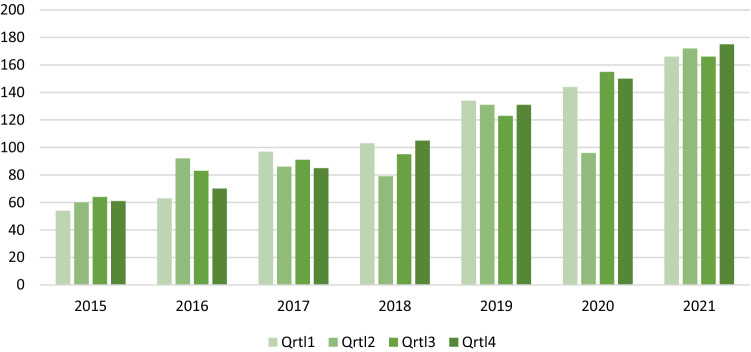
Total number of cases of endometriosis from 2015 to 2021 (outpatient department and surgery)

To evaluate the treatment of patients with endometriosis diagnosed and treated at our specialized centre we analysed the numbers of cases of all treated patients diagnosed with endometriosis quarterly (Fig. 1) and compared them to each preceding year (Table 1).

**Table 1:** Differences in total number of cases of endometriosis quarterly compared to the preceding year

	Number of cases in outpatient department	Number of surgical cases	Total number of cases diagnosed with endo-metriosis	Difference in number of cases to the preceding year-number of cases in outpatient department		Difference in number of cases to the preceding year-number of surgical cases		Difference in number of cases to the preceding year-total number of cases	
				*n*	%	*n*	%	*n*	%
2015									
Qrtl1	38	16	54						
Qrtl2	49	11	60						
Qrtl3	49	15	64						
Qrtl4	45	16	61						
	181	58	239						
2016									
Qrtl1	50	13	63	12	** + 32**	− 3	*− 19*	9	**+ 17**
Qrtl2	66	26	92	17	** + 35**	15	** + 136**	32	** + 53**
Qrtl3	64	19	83	15	** + 31**	4	**+ 27**	19	** + 30**
Qrtl4	56	14	70	11	** + 24**	− 2	*− 13*	9	** + 15**
	236	72	308	55	** + 30**	14	**+ 24**	69	** + 29**
2017									
Qrtl1	78	19	97	28	**+ 56**	6	** + 46**	34	** + 54**
Qrtl2	58	28	86	− 8	*− 12*	2	**+ 8**	− 6	*− 7*
Qrtl3	73	18	91	9	** + 14**	− 1	*− 5*	8	** + 10**
Qrtl4	70	15	85	14	** + 25**	1	** + 7**	15	** + 21**
	279	80	359	43	** + 18**	8	** + 11**	51	**+ 17**
2018									
Qrtl1	78	25	103	0	0	6	** + 32**	6	** + 6**
Qrtl2	58	21	79	0	0	− 7	*− 25*	− 7	*− 8*
Qrtl3	68	27	95	− 5	*− 7*	9	**+ 50**	4	**+ 4**
Qrtl4	77	28	105	7	**+ 10**	13	** + 87**	20	**+ 24**
	281	101	382	2	**1**	21	**+ 26**	23	** + 6**
2019									
Qrtl1	100	34	134	22	**+ 28**	9	** + 36**	31	** + 30**
Qrtl2	99	32	131	41	**+ 71**	11	** + 52**	52	** + 66**
Qrtl3	102	21	123	34	** + 50**	− 6	*− 22*	28	** + 29**
Qrtl4	108	23	131	31	** + 40**	− 5	*− 18*	26	** + 25**
	409	110	519	137	** + 49**	9	** + 9**	137	** + 36**
2020									
Qrtl1	120	24	144	20	** + 20**	− 10	*− 29*	10	** + 7**
Qrtl2	78	18	96	− 21	*− 21*	− 14	*− 44*	− 35	*− 27*
Qrtl3	123	32	155	21	** + 21**	11	** + 52**	32	**+ 26**
Qrtl4	124	26	150	16	** + 15**	3	** + 13**	19	**+ 15**
	445	100	545	36	**+ 9**	− 10	*− 9*	26	** + 5**
2021									
Qrtl1	128	38	166	8	** + 7**	14	**+ 58**	22	** + 15**
Qrtl2	132	40	172	54	** + 69**	22	** + 122**	76	** + 79**
Qrtl3	99	67	166	− 24	*− 20*	35	** + 109**	11	**+ 7**
Qrtl4	119	56	175	− 5	*− 4*	30	**+ 115**	25	** + 17**
Total	478	201	679	33	** + 7**	101	** + 101**	134	** + 25**
Total 7 Years	2309	722	3031						

Throughout the 5-year period before the COVID-19 pandemic a continuous increase in the number of cases in the outpatient department as well as in surgical cases can be found with only short intervals with little decreases, reaching its peak in 2019, compare to 2018 with nearly 50% more contacts in the outpatient department (409 contacts in absolute numbers vs. 281) and + 36% in the total number of cases (519 vs 382 patients).

Focussing on the last 3 years, visits due to endometriosis in our outpatient department increased over 50% from 36 to 55 in January 2020 compared to January 2019, followed by an ongoing decrease for consultations in the period from March to May (− 25%, − 37%, − 23%). During the first lock down phase between 03/22/2020 and 05/05/2020 9 patients were treated in outpatient department due to endometriosis, 36 were treated in the same period in 2019 (*p* < 0.001). Another decline in patient numbers was seen in August (− 4%) and November 2020 (− 7%) during the so-called second wave (Table 2) [20]. In 2021, the second year of the COVID-19 pandemic, a little decrease in the numbers of cases treated in the outpatient department was found in February and May and a stronger decrease in August and September (− 15% and − 8%) and in November 2021 (− 5%).

**Table 2:** Number of cases in outpatient department, 2020 and 2021 compared to 2019

Number of cases in outpatient department	2019	2020			2021		
			Difference in number of cases compared to 2019			Difference in number of cases compared to 2019	
			*n*	%		*n*	%
Jan	36	55	19	**53**	52	16	**44**
Feb	36	44	8	**22**	35	− 1	*− 3*
Mar	28	21	− 7	*− 25*	41	13	**46**
Apr	41	26	− 15	*− 37*	52	11	**27**
May	35	27	− 8	*− 23*	31	− 4	*− 11*
Jun	23	25	2	**9**	49	26	**113**
Jul	34	54	20	**59**	54	20	**59**
Aug	25	21	− 4	*− 16*	10	− 15	*− 60*
Sep	43	48	5	**12**	35	− 8	*− 19*
Oct	45	68	23	**51**	54	9	**20**
Nov	42	35	− 7	*− 17*	37	− 5	*− 12*
Dec	21	21	0	0	28	7	**33**
Total	409	445	36	**9**	478	69	**17**

**Fig. 2:**
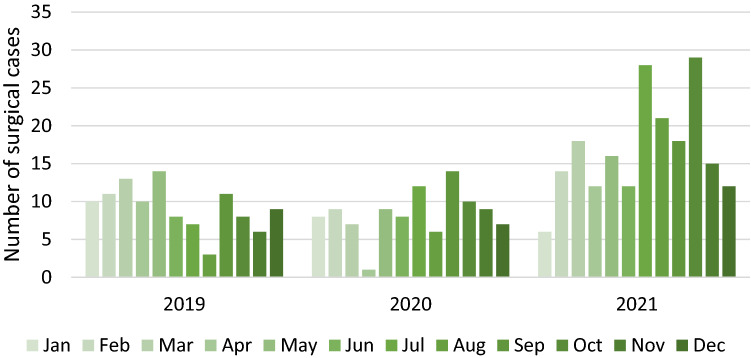
Number of surgical cases from 2019 to 2021 showing a decrease during lockdown period in 2020 and an increase in 2021

Comparing the number of surgical cases from 2020 and 2021 to those from 2019, a decrease in surgeries for endometriosis especially in the first half of 2020 can be found. The first lock down phase in Germany significantly affected the number of surgeries with a relevant decline in March (− 46%), April (− 90%) and May (−  36%). Looking at the exact patient numbers between 03/22/2020 and 05/05/2020 and comparing it to the same period in 2019 a significant reduction was found (27 vs. 52 patients treated surgically, *p* < 0.001). In contrast, during the second half of the year the number of surgical cases of endometriosis increased, e.g., by + 71% in July and + 100% in August (Table 3, Fig. 2).

**Table 3:** Number of surgical cases, 2020 and 2021 compared to 2019

Number of surgical cases	2019	2020			2021		
			Difference in number of cases compared to 2019			Difference in number of cases compared to 2019	
			*n*	%		*n*	%
Jan	10	8	− 2	*− 20*	6	− 4	*− 40*
Feb	11	9	− 2	*− 18*	14	3	**27**
Mar	13	7	− 6	*− 46*	18	5	**38**
Apr	10	1	− 9	*− 90*	12	2	**20**
May	14	9	− 5	*− 36*	16	2	**14**
Jun	8	8	0	0	12	4	**50**
Jul	7	12	5	**71**	28	21	**300**
Aug	3	6	3	**100**	21	18	**600**
Sep	11	14	3	**27**	18	7	**64**
Oct	8	10	2	**25**	29	21	**263**
Nov	6	9	3	**50**	15	9	**150**
Dec	9	7	− 2	*− 22*	12	3	**33**
Total	110	100	− 10	*− 9*	201	91	**83**

Similar to the second wave at the end of 2020, we found another reduction of cases in December 2020 (− 22%) and in January 2021 (− 40%). In the following months the numbers rose again with a peak in July and August with an increase of 300% and 600% (July 2019 vs. 2021: 7 vs. 21 patients, August 2019 vs. 2021: 3 vs. 21 patients; Table 3).

Taken together, the detected decrease in the first half of 2020 resulted in an overall reduction in surgery for endometriosis by − 9%, which could be compensated in 2021, with an increase of + 83% in comparison to 2019 can be found (Table 3).

Comparing The comparison of treated patients (conservatively and surgically) from 2019 reveals an increase of cases by 5% in 2020 and 31% in 2021 despite the COVID-19 pandemic (519 patients (2019) vs. 545 patients (2020) vs. 679 patients (2021); Table 4).

**Table 4:** Total number of cases diagnosed with endometriosis, 2020 and 2021 compared to 2019

Total number of cases diagnosed with endometriosis	2019	2020			2021		
			Difference in number of cases compared to 2019—total number of cases			Difference in number of cases compared to 2019—total number of cases	
			*n*	%		*n*	%
Jan	46	63	17	**37**	58	12	**26**
Feb	47	53	6	**13**	49	2	**4**
Mar	41	28	− 13	*− 32*	59	18	**44**
Apr	51	27	− 24	*− 47*	64	13	**25**
May	49	36	− 13	*− 27*	47	− 2	*− 4*
Jun	31	33	2	**6**	61	30	**97**
Jul	41	66	25	**61**	82	41	**100**
Aug	28	27	− 1	*− 4*	31	3	**11**
Sep	54	62	8	**15**	53	− 1	*− 2*
Oct	53	78	25	**47**	83	30	**57**
Nov	48	44	− 4	*− 8*	52	4	**8**
Dec	30	28	− 2	*− 7*	40	10	**33**
Total	519	545	26	**5**	679	160	**31**

After a partial decrease in COVID-19 pandemic due to restrictions in outpatient department and OR capacities patient contacts were rising again in 2021. This led to a threefold multiplication of numbers in 2021 on the outpatient base as well as surgeries compared to 2015 (Surgery: 72 vs. 201 patients, outpatient department: 181 vs. 478 patients).

## Discussion

In an overall view, we see a remarkable increase in cases treated for endometriosis within the years even including the slight decrease due to the pandemic in 2020.

Several hypotheses for the overall increase of patients treated for endometriosis during the observed interval can be stated. Patients` information status about the disease improved and consequently the demand for facilities specialized on endometriosis treatment increased. Especially the certification as a specialized centre for the treatment of endometriosis in March 2013 can be seen as important factor. With the certification of the endometriosis centre at the highest level by SEF in 2019 a further impact could be generated. The capacities in the outpatient as well as the surgical setting were increased, the visibility and publicity of the centre was improved by a better web appearance and by regular educational offers to the cooperating doctors [21].

Endometriosis per se is known to be the “disease without a lobby” and is not classified as life threatening. Therefore, less money is invested and less research is done than for malignant diseases [22]. However, platforms for self-help and initiatives such as “Endometriose-Vereinigung Deutschland e. V.” and “Stiftung Endometriose Forschung” in Germany and the European Information Centre reach more and more patients suffering from symptoms described above and help to create a better public awareness. The increased usage of online information for patients supports them in finding a specialized centre in addition to interpersonal communication. Literature is still very heterogeneous, but around 33% of patients with chronic pelvic pain are estimated to suffer from this disease [23, 24]. It is unlikely that the prevalence of endometriosis has increased but rather that the awareness in patients and health care providers has.

Although per se not life-threatening symptoms of endometriosis are a big burden for patients and lead to a reduced quality of life [16]. Nowadays, better informed patients want to know more about the disease and want to exclude that it is the reason for their pain.

Still, the diagnosis of endometriosis can only be made via laparoscopy with a histopathological correlate [24]. However, more and more specialists recommend early endocrine therapy and consider the diagnosis confirmed by the reduction of pain. This is even more important in times when anaesthesia and surgery are to be avoided to minimize the risk for patients and health care providers in situations, such as the COVID-19 pandemic [25].

Focusing on the interval since the beginning of the COVID-19 pandemic, this analysis of cases with endometriosis treated at the Department of Obstetrics and Gynaecology of the University Hospital, LMU Munich, shows a severe decrease of cases treated for endometriosis on an outpatient as well as inpatient base in 2020 synchronic to the first COVID-19-wave and respective lockdown period in comparison with those of 2019.

Another decrease at the end of 2020 can be detected and attributed to the implications and restrictions caused by the second wave [20].

Due to pandemic restrictions to reserve capacities for COVID-patients with the need for inpatient treatment the regular outpatient contacts of our endometriosis centre were limited to severe or emergency cases. This was also the case for the surgical capacities. Only patients with severe symptoms were appointed for surgery.

Treatment approaches with primary endocrine therapy were preferred in these situations.

In 2021, the second year of the COVID-19 pandemic the number of cases treated in the outpatient department was reduced as a result of implications of the third and the fourth wave.

The number of surgical cases with endometriosis was reduced in the first lockdown period resulting in an overall reduction in 2020 in comparison to 2019. In contrast in 2021 this reduction could be compensated. The number of patients with endometriosis treated with surgery showed an increase of + 83% in comparison to 2019 symbolizing a health care system that found its adaptation to pandemic circumstances.

Endometriosis has strong implications on fertility. In times of COVID-19, there are signs that couples are focusing more on building a family. Fertility centres seem to have to face more patients and have a higher demand [26], which could also be a factor for greater importance of endometriosis treatment during or despite the COVID-19 pandemic. Furthermore, Rosielle et. al showed that especially endometriosis and infertility patients self-reported high levels of stress during the COVID-19 pandemic [19].

Contrarily Schwab et al. showed that on the one hand patients diagnosed with endometriosis showed decreased levels of physical pain and disability during the first lockdown in March 2020. On the other hand an increase of emotional stress and subjectively less support from family and friends was reported [27]. This, could be a reason for a higher demand in outpatient departments after the lockdown period, too, when non-emergency visits were possible again.

In summary, the quantity of patients treated for endometriosis at our high-level centre specialized for endometriosis at the Department of Obstetrics and Gynaecology of the University Hospital LMU Munich increased constantly over the last 7 years pointing to a new awareness for the disease with an accompanying improvement in treatment. Focusing on the COVID-19 pandemic, the number of cases declined in the first lockdown period. The well-structured management and organization in our endometriosis centre, which is attributable to the requirements of the SEF certification process, helped that the numbers increased right away significantly after the lockdown nevertheless, leading to a delay, but not an absence of treatment.

A limitation of our investigation is its design. As a descriptive analysis, we can only hypothesize the causes that lead to this development of patient numbers. Furthermore, as the COVID-19 pandemic is still ongoing, there might be changes over time. Further research needs to be done on benign diseases and their treatment during the COVID-19 pandemic, since also benign diseases can seriously impact patients´ lives.

## Conclusions

In our analysis, we found rising numbers of treated patients with endometriosis through the last 7 years, which can be a symbol for a growing public awareness towards endometriosis in general. In particular, it underlines the importance and success of certified centres as is the case for the centre of endometriosis at the LMU Department of Obstetrics and Gynaecology leading to an improvement of treatment as well as quality of life of patients suffering from endometriosis.

But the COVID-19 pandemic had and has high implications for patients and health care systems worldwide. Although significantly fewer patients could be treated during the lockdown period the overall number increased due to a rapid increase afterwards. Consequently, the increase in cases requires more attention and a further standardised approach to enhance treatment with the consequence of better health conditions as well as quality of life of those affected.

Further studies need to be done to evaluate the ongoing impact of COVID-19 on the health care sector.
